# Effect of Cobalt-Based Filler Wire Composition on the Microstructure and High-Temperature Properties of Cladding Layers on Ni-Based Superalloy

**DOI:** 10.3390/ma19143090

**Published:** 2026-07-17

**Authors:** Shuai Huang, Tianyuan Wang, Wei Liu, Yu Wu, Jian Miao, Guohui Zhang, Bingqing Chen, Biao Zhou

**Affiliations:** 3D Printing Research and Engineering Technology Center, Beijing Institute of Aeronautical Materials, Beijing 100095, China

**Keywords:** DD5 single crystal superalloy, cobalt-based filler wire, cladding layer, high-temperature tribological behavior, high-temperature tensile properties

## Abstract

To improve the high-temperature service performance of cladding layers on DD5 single crystal superalloy, this study comparatively investigated the effects of two cobalt-based filler wires, PMet931 and PMet994, on the microstructural evolution, hardness, high-temperature tensile properties, and friction and wear behavior of the cladding layers. The results show that PMet931, with a higher Ni content, exhibits better compositional compatibility and interfacial metallurgical compatibility with the DD5 Ni-based substrate. In contrast, the higher W, C, and Cr contents in PMet994 promote the formation of W/Cr-rich secondary phases and grain refinement, resulting in higher hardness and better high-temperature strength retention. Both filler wires can form continuous cladding layers on the DD5 surface. The PMet931 cladding layer shows a more homogeneous microstructure and a smoother interfacial transition, whereas the PMet994 cladding layer contains more secondary phases and exhibits more pronounced strengthening features. Mechanical testing indicates that PMet931 provides a better strength ductility balance at room temperature, while PMet994 shows higher strength and hardness retention over the range of 800–1050 °C, with a tensile strength at 1050 °C approximately 53% higher than that of PMet931. The friction and wear results show that the wear rate of the PMet994 cladding layer was significantly lower than that of the PMet931 cladding layer at 800 °C, whereas the difference between the two cladding layers decreased at higher temperatures. This study demonstrates that filler wire composition significantly affects the high-temperature performance of DD5 cladding layers by regulating secondary phase precipitation, interfacial compatibility, and microstructural stability.

## 1. Introduction

Ni based single crystal superalloys are widely used in hot section components of aero engines, such as turbine blades [1] and guide vanes [2], owing to their excellent high-temperature strength, creep resistance, and oxidation resistance [3,4]. As a typical second-generation Ni based single crystal superalloy, DD5 exhibits good microstructural stability and service reliability under high-temperature loading and complex thermomechanical coupling conditions [5]. However, during long term service, the surfaces of the hot section components are subjected to multiple factors, including high-temperature gas erosion, contact wear, oxidation corrosion, and cyclic thermal stress. As a result, local damage such as wear, spalling, cracking, and dimensional deviation is prone to occur [6,7]. Due to the high manufacturing cost and long processing cycle of single crystal superalloy components, surface repair and strengthening of locally damaged regions [8] are of great significance for extending service life, reducing maintenance costs, and improving aero engine reliability [9].

Cladding can produce an alloy layer with a specific composition and function on the substrate surface and is an important method for restoring the geometry of hot section components and improving their surface service performance [10]. For Ni based single crystal superalloys such as DD5, an ideal cladding layer should not only possess high hardness, high-temperature strength, and wear resistance but also form a stable metallurgical bonding interface with the substrate [11]. Thereby, this avoids interfacial cracking or premature failure caused by abrupt compositional changes, microstructural discontinuity, and differences in thermophysical properties [12,13].

Cobalt-based alloys have good high-temperature strength, oxidation resistance, and wear resistance and are commonly used as cladding materials for high-temperature surface strengthening and wear protection [14]. Their properties are closely related to the contents of alloying elements such as Cr, W, C, and Ni, as well as the formation of secondary phases such as carbides [15] and intermetallic compounds [16]. Among these elements, Cr generally improves oxidation and corrosion resistance [17], W enhances high-temperature strength through solid solution strengthening [16], C readily combines with strong carbide forming elements to form hard phases, and Ni may affect the compositional compatibility with Ni based substrates [15]. Therefore, cobalt-based filler wires with different compositions may form different solidification microstructures, interfacial diffusion characteristics, and high-temperature performance responses after cladding on Ni based single crystal superalloys [18].

The microstructure and properties of cladding layers are jointly controlled by multiple factors, including filler wire composition, molten pool solidification behavior, substrate dilution, and interfacial elemental diffusion [19]. During cladding, elements such as Co, Cr, W, and C in the filler wire inter diffuse with elements such as Ni, Al, Ta, and Re in the DD5 substrate, accompanied by local segregation [20]. This further changes the phase constitution, grain morphology, secondary phase distribution, and interfacial bonding state in the fusion zone [21]. In particular, the DD5 substrate has a typical γ/γ′ two phase microstructure and a strict single crystal orientation. The thermal cycles during cladding may disturb the original microstructural stability near the interface and induce polycrystallization, dendritic growth, or enrichment of strengthening phases [22]. At present, studies on cobalt-based cladding layers mainly focus on process optimization, wear resistance, or microstructural characterization of a single alloy system. However, systematic comparative studies on the microstructural evolution, elemental diffusion, and high-temperature performance differences of cobalt-based filler wires with different compositions after cladding on DD5 single crystal superalloy remain limited.

Based on this, two cobalt-based filler wires with different compositions, PMet931 and PMet994, were selected to prepare cladding layers on the surface of DD5 single crystal superalloy, and the effect of filler wire composition on the microstructure and high-temperature properties of the cladding layers was investigated. First, the chemical composition, phase constitution, microstructure, and hardness of the two filler wires were characterized. Then, the microstructure, interfacial elemental diffusion, and grain structure characteristics of the two cladding layers were analyzed by SEM, EDS, and EBSD. Finally, hardness testing, room-temperature and high-temperature tensile tests, and high-temperature friction and wear tests were conducted to reveal the differences between the cladding layers produced with cobalt-based filler wires of different compositions in terms of strength ductility balance, high-temperature strength retention, and wear resistance. The relationship among filler wire composition, interfacial microstructure, strengthening mechanism, and high-temperature properties was established, providing a reference for surface material selection and cladding layer performance optimization of DD5 single crystal superalloy hot section components.

## 2. Experimental Methods and Equipment

### 2.1. Experimental Materials and Methods

In this study, DD5 single crystal superalloy plates were selected as the substrate material, while PMet931 and PMet994 cobalt-based superalloy filler wires were used as the cladding materials. The chemical compositions of the DD5 substrate and the two cobalt-based filler wires are listed in Table 1. Prior to cladding, the DD5 substrate was machined into plate specimens with dimensions of 100mm×50mm×5mm and the surfaces to be cladded were mechanically ground and cleaned with anhydrous ethanol to remove surface oxide films, oil contamination, and other impurities. PMet931 and PMet994 cobalt-based cladding layers were deposited on the DD5 surface using argon arc cladding. During the cladding process, high purity argon was used as the shielding gas. The main processing parameters were a cladding current of 32 A, an arc voltage of 16 V, a travel speed of 0.5 mm/s, a wire feeding rate of 3 mm/s, and a filler wire diameter of 1.6 mm. The shielding gas flow rate was 10 L/min. During cladding, the torch angle was maintained at approximately 70° relative to the substrate surface, and the distance between the tungsten electrode tip and the substrate surface was approximately 2 mm. After cladding, the specimens were naturally cooled to room temperature in air. The specimens prepared using PMet931 and PMet994 filler wires were designated as PMet931/DD5 and PMet994/DD5, respectively. For each filler wire, three independently cladded specimens were prepared under the same processing conditions. Subsequently, cross-sectional specimens were cut perpendicularly to the welding direction for microstructural characterization, interfacial elemental diffusion analysis, hardness testing, tensile testing, and high-temperature friction and wear testing. The specimens used for these tests were obtained from independently produced cladded specimens to ensure the reliability and repeatability of the experimental results.

### 2.2. Microstructural Characterization Methods

To analyze the intrinsic microstructural characteristics of PMet931 and PMet994 cobalt-based filler wires and their microstructural evolution behavior after cladding on DD5 single crystal superalloy, X-ray diffraction (XRD, D8 Discover, Bruker, Billerica, MA, USA), scanning electron microscopy (SEM, Sigma, ZEISS, Oberkochen, Germany), energy dispersive spectroscopy (EDS, X-Max N, Oxford Instruments, Abingdon, UK), and electron backscatter diffraction (EBSD, NordlysMax^2^, Oxford Instruments, Abingdon, UK) characterizations were performed on both the as-received filler wires and the cladded specimens. An X-ray diffractometer was used to analyze the phase constitution of the PMet931 and PMet994 filler wires and their corresponding cladding layers. For XRD analysis of the filler wires, the PMet931 and PMet994 wire samples were cut, mounted, ground, and mechanically polished to obtain flat testing surfaces. The XRD measurements were performed on the polished flat surfaces of the filler wire specimens rather than directly on the curved wire surfaces. During the tests, a Cu target radiation source was employed with a scanning angle range of 10–90° to identify the major phases present in the two filler wires and their cladding layers [23]. Owing to the differences in alloying element contents between the two filler wires, variations in diffraction peak positions and intensities can be used to assist in determining the phase constitution and strengthening phase characteristics of the cladding layers.

Before microstructural observation, cross-sectional specimens were cut perpendicular to the welding direction using wire electrical discharge machining. The specimens were sequentially subjected to coarse grinding, fine grinding, and mechanical polishing, followed by chemical etching to reveal the microstructures of the cladding layer, fusion zone, and DD5 substrate. The microstructures of different regions in the PMet931/DD5 and PMet994/DD5 cladded specimens, including the cladding layer, fusion zone, heat affected zone, and substrate, were observed using scanning electron microscopy. Particular attention was paid to dendritic morphology, secondary phase distribution, interfacial bonding state, and microstructural changes near the interface. Energy dispersive spectroscopy was used to characterize the elemental distribution in the as-received filler wires and cross-sectional regions of the cladded specimens. For the cladded specimens, EDS mapping and point analysis were mainly performed near the cladding layer/substrate interface to analyze the distribution characteristics of the major alloying elements such as Co, Ni, Cr, and W across the cladding layer, fusion zone, and DD5 substrate, thereby clarifying the elemental diffusion and compositional transition behaviors formed during cladding with different filler wires.

Electron backscatter diffraction was used to analyze the grain structure and orientation characteristics of the two filler wires and their cladding layers. Before EBSD testing, the specimens were mechanically polished and then further finely polished to reduce the influence of the surface deformation layer on orientation indexing. The differences in grain structure between PMet931 and PMet994 filler wires and their cladding layers were analyzed using inverse pole figure maps, grain boundary distribution maps, grain size distributions, and misorientation statistics. The grain misorientation angle was defined as the minimum crystallographic rotation angle between neighboring grains or adjacent orientation regions in the EBSD dataset. It was not calculated with respect to a fixed external reference direction. A misorientation angle of 0° represents identical or nearly identical crystallographic orientations. To reduce the influence of EBSD orientation noise, misorientation angles below 2° were not considered in the grain boundary statistics. In the grain boundary analysis, boundaries with misorientation angles of 2–15° were defined as low-angle grain boundaries, while those with misorientation angles greater than 15° were defined as high-angle grain boundaries.

### 2.3. Mechanical Properties and Friction and Wear Testing

To evaluate the mechanical properties and high-temperature tribological behavior of PMet931 and PMet994 cobalt-based filler wires after cladding on DD5 single crystal superalloy, microhardness testing, tensile testing at 25, 800, 900, 1000, and 1050 °C, and high-temperature friction and wear testing were conducted on the two cladded specimens. A Vickers microhardness tester was used to measure the hardness of the cross sections of the PMet931/DD5 and PMet994/DD5 cladded specimens. The measurement locations were arranged along the direction from the cladding layer to the DD5 substrate, sequentially covering the cladding layer, fusion zone, and substrate regions to obtain the hardness distribution characteristics of different regions. Multiple measurement points were selected in each region, and the average value was taken as the microhardness result for that region. The hardness tests were conducted in accordance with GB/T 4340.1-2009 [24].

**Figure 1:**
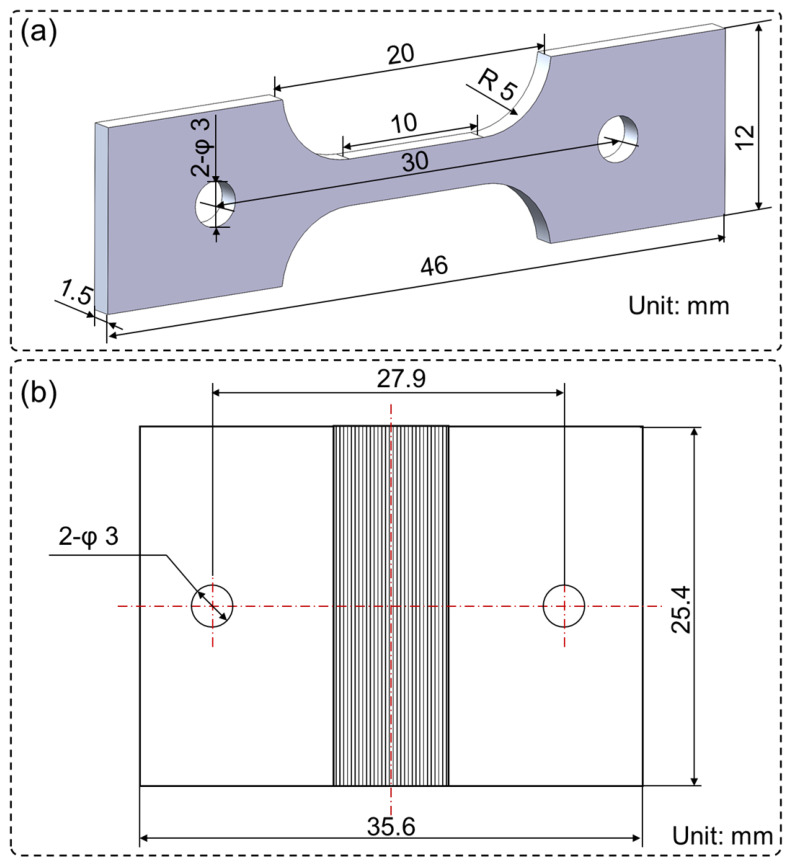
Tensile, friction, and wear test specimens: (**a**) geometry of the tensile specimen; (**b**) specimen for friction and wear testing.

Room-temperature and high-temperature tensile tests were conducted to evaluate the load-bearing capacity and temperature sensitivity of the two cladded specimens. Tensile specimens were machined from the cladded DD5 plates, ensuring that the cladding layer was located within the effective gauge section of the specimen, as shown in Figure 1a. The tensile tests were performed at 25, 800, 900, 1000, and 1050 °C. For the high-temperature tensile tests, the specimens were heated to the target temperature and held for 10 min to ensure a uniform temperature distribution before loading. Stress–strain curves were recorded during the tensile tests, and the ultimate tensile strength, yield strength, and elongation were obtained. For each cladding layer and each testing temperature, three parallel specimens were tested under identical conditions, and the average values were reported.

A Bruker UMT TriboLab multifunctional tribometer (Bruker, Billerica, MA, USA) was used to evaluate the tribological behavior of the two cladding layers under high-temperature contact conditions in accordance with ASTM G133-22 [25]. The specimen geometry is shown in Figure 1b. The friction and wear tests were conducted at 800, 900, and 1000 °C. The lower specimen had a thickness of 8.9 mm, a length of 35.6 mm, and a width of 25.4 mm, with a rectangular cladding region measuring 15 mm in width and 1 mm in depth. The upper specimen was a silicon nitride ball with a diameter of 6 mm, which was used as the counterface material. The tests were conducted under a normal load of 20 N, a stroke length of 5 mm, a frequency of 5 Hz, and a sliding duration of 30 min. Before testing, the specimens were heated to the target temperature and held for sufficient time to ensure temperature stability. During testing, the coefficient of friction (COF) as a function of time was continuously recorded. After testing, the wear scar morphology was observed, and the wear volume was measured. The wear rate was calculated according to the wear volume, normal load, and total sliding distance. To further analyze the wear mechanisms, the wear scar surface morphology was observed using scanning electron microscopy, combined with EDS analysis of elemental distribution in the wear region.

## 3. Results and Discussion

### 3.1. Microstructure and Basic Properties of Cobalt-Based Filler Wires

#### 3.1.1. Composition, Phases, and Hardness of the Filler Wires

To clarify the effects of different cobalt-based filler wires on the microstructure and high-temperature properties of cladding layers on DD5 single crystal superalloy, the chemical composition, phase constitution, and initial hardness of PMet931 and PMet994 were first comparatively analyzed. The alloying element contents of the filler wires not only determine the solidification microstructure and types of strengthening phases in the deposited metal but also affect elemental diffusion behavior, interfacial metallurgical bonding, and subsequent high-temperature service performance when cladded on a Ni based single crystal superalloy substrate.

As shown in Table 1, both PMet931 and PMet994 are Co-based alloy filler wires containing relatively high levels of Cr, W, and Ni, whereas their alloying designs differ significantly. The contents of Cr, W, and C in PMet994 are higher than those in PMet931. In particular, the W content increases from 7.37 wt% in PMet931 to 18.80 wt% in PMet994, and the C content increases from 0.46 to 0.95 wt%. W is an important solid solution strengthening element in cobalt-based superalloys, which can improve the high-temperature strength and thermal stability of the matrix. C promotes the formation of carbide strengthening phases with elements such as Cr and W, thereby enhancing the resistance to plastic deformation and high-temperature wear. Therefore, the higher W and C contents in PMet994 may provide an important compositional basis for its higher hardness and high-temperature strength. In contrast, the Ni content in PMet931 is 10.61 wt%, which is markedly higher than that in PMet994, which is 5.40 wt%. Since DD5 is a Ni based single crystal superalloy, the higher Ni content may help to improve the metallurgical compatibility between the filler wire and the substrate, reducing microstructural incompatibility caused by abrupt compositional changes during cladding.

**Figure 2:**
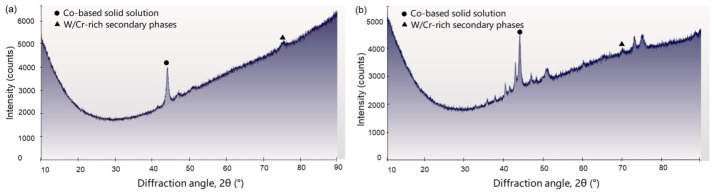
Indexed XRD patterns of PMet931 and PMet994 filler wires: (**a**) PMet931; (**b**) PMet994. The diffraction peaks were assigned by comparison with standard reference diffraction patterns.

Figure 2 shows the indexed XRD patterns of the PMet931 and PMet994 filler wires. The main diffraction peaks of both filler wires can be assigned to a Co-based solid solution, indicating that the matrices of the two filler wires are primarily composed of a Co-based solid solution. Compared with PMet931, PMet994 exhibits additional weak diffraction features and more pronounced peak profile variations, suggesting a higher tendency for secondary phase formation. These features are consistent with the higher W, C, and Cr contents in PMet994 and the SEM/EDS observations, where more W/Cr-rich secondary phases were detected. Considering the weak intensity and partial overlap of minor diffraction peaks, these phases are conservatively described as W/Cr-rich secondary phases rather than being directly identified as carbides.

**Figure 3:**
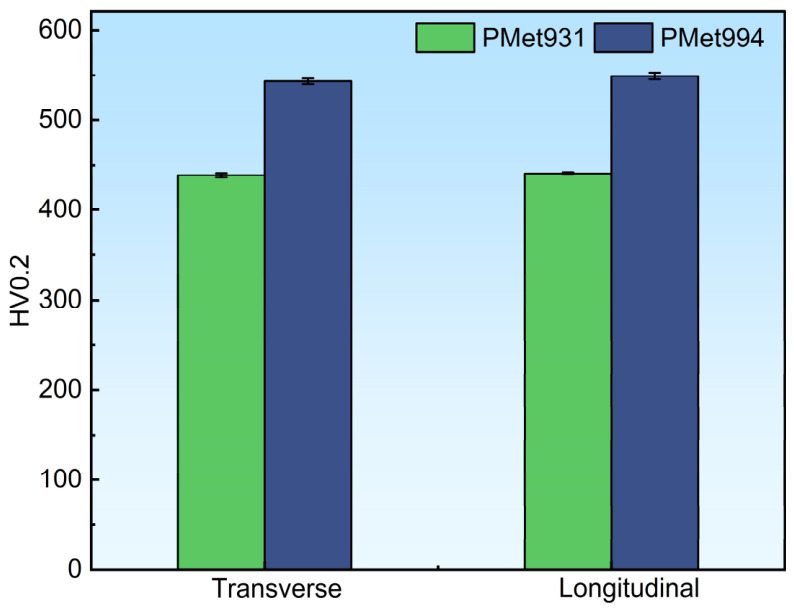
Comparison of transverse and longitudinal Vickers hardness of PMet931 and PMet994 filler wires.

Figure 3 presents the Vickers hardness values of the two filler wires measured in the transverse and longitudinal directions. It can be seen that the transverse and longitudinal hardness values of the PMet931 filler wire are both approximately 440 HV0.2, whereas those of the PMet994 filler wire are approximately 543 HV0.2 and 549 HV0.2, respectively, which are significantly higher than those of PMet931. Based on the average hardness values, the hardness of PMet994 is approximately 546 HV0.2, while that of PMet931 is approximately 440 HV0.2, indicating an increase of approximately 24% for PMet994 compared with PMet931. In addition, only minor differences are observed between the transverse and longitudinal hardness values of both filler wires, indicating a relatively uniform hardness distribution without obvious directional dependence. The higher hardness of PMet994 is mainly attributed to its higher W, C, and Cr contents. Among these elements, W enhances the matrix strength through solid solution strengthening, while the carbide phases formed by the combination of C with elements such as Cr and W further improve the hardness and wear resistance of the material.

In summary, although both PMet931 and PMet994 are cobalt-based filler wires, they exhibit significant differences in alloy design and intrinsic properties. PMet931 contains a higher Ni content, which may be more favorable for forming good metallurgical bonding with the Ni based DD5 alloy. In contrast, PMet994 contains higher levels of W, C, and Cr, resulting in higher initial hardness and potentially superior high-temperature strengthening capability. Therefore, after cladding on the surface of DD5 single crystal superalloy, the two filler wires are expected to exhibit different characteristics in terms of solidification microstructure, strengthening phase distribution, interfacial elemental diffusion behavior, and high-temperature mechanical and tribological properties. These differences will be further analyzed in combination with the microstructural characterization and high-temperature performance results of the cladding layers.

#### 3.1.2. Microstructure of the As-Received Filler Wires

**Figure 4:**
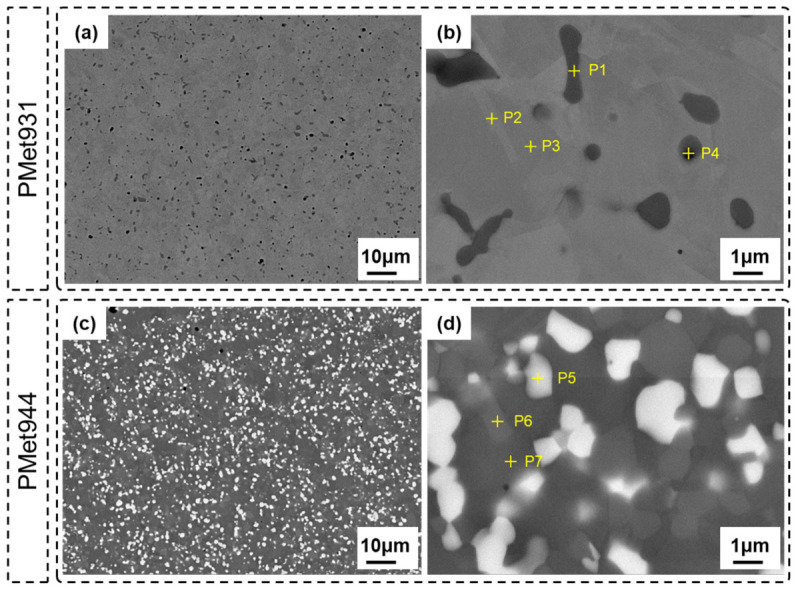
SEM microstructures of the filler wires: (**a**,**b**) PMet931; (**c**,**d**) PMet994.

Figure 4 shows the SEM microstructures and corresponding EDS point analysis results of the PMet931 and PMet994 filler wires. Both filler wires consist of a Co-based solid solution matrix and dispersed secondary phase particles, whereas significant differences exist in the quantity, size, and distribution of the secondary phases. The microstructure of the PMet931 filler wire is relatively homogeneous overall. Under low magnification, a small number of black or dark gray particulate secondary phases can be observed, with relatively low particle density and sparse distribution. Under high magnification, irregular particles or short rod like phases are present in local regions, exhibiting relatively large sizes and discontinuous distribution. In contrast, the number of secondary phase particles in the PMet994 filler wire increases markedly. Under low magnification, a large number of bright white particles can be observed uniformly dispersed throughout the matrix. At higher magnification, these particles mainly exhibit blocky, near spherical, or irregular polygonal morphologies, indicating more pronounced secondary phase precipitation characteristics in the PMet994 filler wire.

The EDS analysis results are listed in Table 2 and the elemental compositions of different microregions in PMet931 vary considerably. Some particle regions show enrichment of W and Cr, indicating the possible formation of W rich or Cr rich secondary phases. Overall, however, the number of secondary phases in PMet931 is relatively low, and the matrix region is still mainly composed of Co, Cr, and Ni. For PMet994, the bright white particle regions exhibit a higher W content, accompanied by certain enrichment of Cr and Co, suggesting that these secondary phases are mainly W/Cr-rich secondary phases. This result corresponds well to the higher W, C, and Cr contents in the PMet994 filler wire, indicating that a high content of strong carbide forming elements promotes the precipitation and dispersion of secondary phases. The presence of abundant hard secondary phases can increase the resistance to the dislocation motion and enhance the strengthening effect of the matrix, which is an important microstructural reason for the significantly higher hardness of PMet994 than PMet931.

**Figure 5:**
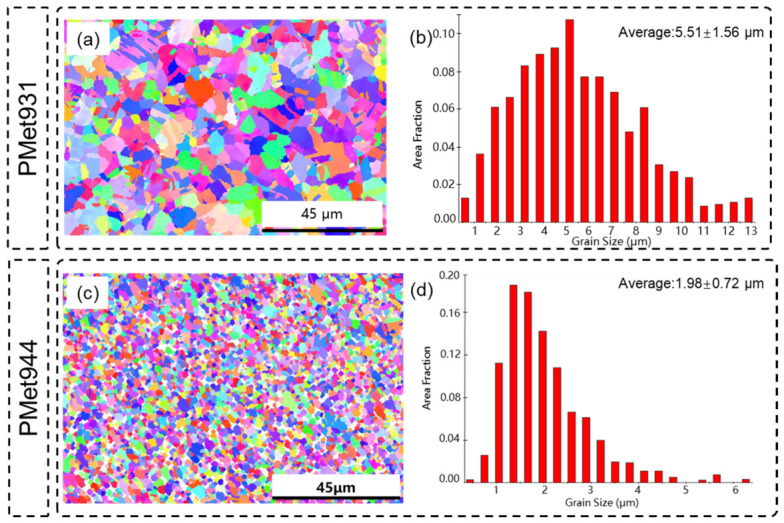
EBSD orientation maps and grain size distributions of the filler wires: (**a**,**b**) PMet931; (**c**,**d**) PMet994.

Figure 5 shows the EBSD orientation maps and grain size distributions of the two filler wires. The EBSD results indicate that both PMet931 and PMet994 exhibit polycrystalline orientation characteristics with relatively random orientation distributions and no obvious preferred crystallographic texture. However, significant differences in grain size are observed between the two filler wires. The grain size of PMet931 is relatively large, with the microstructures mainly consisting of equiaxed grains and locally elongated grains. The grain size distribution spans a relatively wide range, indicating comparatively lower microstructural uniformity. In contrast, PMet994 exhibits evident grain refinement, with a larger number of grains and a more concentrated grain size distribution, showing an overall fine equiaxed grain structure. The grain refinement observed in PMet994 may be related to the higher contents of W, C, and Cr. On the one hand, alloying elements such as W and Cr can enhance the degree of solid solution strengthening of the matrix and reduce the grain boundary mobility during microstructural evolution. On the other hand, a large number of dispersed secondary phase particles can exert a pinning effect on the grain boundary motion, thereby suppressing grain growth. Consequently, PMet994 simultaneously exhibits characteristics of “high secondary phase content” and a “fine grained microstructure”.

According to the mechanisms of grain refinement strengthening and secondary phase strengthening, fine grains increase the grain boundary area and hinder the dislocation slip, while dispersed strengthening phases further improve resistance to plastic deformation. The combined effect of grain refinement and secondary phase strengthening gives the as-received PMet994 filler wire a higher hardness than the as-received PMet931 filler wire.

In summary, PMet931 and PMet994 exhibit obvious differences in microstructure and grain structure. PMet931 contains fewer secondary phase particles and relatively larger grains, resulting in a relatively limited strengthening effect. In contrast, PMet994 contains more dispersed W/Cr-rich secondary phase particles and a finer grain structure, showing stronger secondary phase strengthening and grain refinement strengthening characteristics. These microstructural differences are consistent with the above composition and hardness results, indicating that the higher W, C, and Cr contents in PMet994 are important reasons for its higher hardness and potential high-temperature strengthening capability. These results also provide a basis for the subsequent analysis of microstructural evolution and high-temperature performance differences in DD5 based cladding layers.

### 3.2. Microstructural Characteristics of Cladding Layers Deposited on the DD5 Substrate

To further analyze the effects of cobalt-based filler wires with different compositions on the formation of cladding layer microstructures on DD5 single crystal superalloy, PMet931 and PMet994 filler wires were used to deposit cladding layers on the surface of the DD5 substrate, and the macro-morphology, microstructure, interfacial elemental diffusion, and grain structure of the cladding layers were characterized.

**Figure 6:**
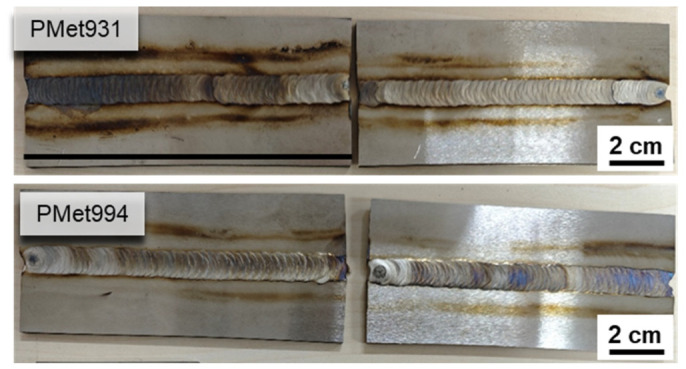
Macro-morphologies of cladding layers deposited on the DD5 substrate surface.

Figure 6 shows the macro-morphologies of the two filler wires after cladding on the DD5 substrate surface. It can be seen that both filler wires are capable of forming continuous cladding layers on the DD5 surface, with overall sound weld-bead formation and without obvious macro-cracks or large-scale delamination defects. The PMet931 cladding layer exhibits relatively continuous ripple patterns on the surface, a comparatively uniform bead width, and a smoother edge transition. The PMet994 cladding layer also shows good continuity; however, more pronounced local surface undulations and larger color variations are observed in some regions, indicating possible differences in molten pool flow behavior, solidification behavior, and surface oxidation state during cladding compared with PMet931. Overall, both cobalt-based filler wires can achieve metallurgical bonding with the DD5 substrate, providing a basis for the subsequent microstructural and property analyses.

**Figure 7:**
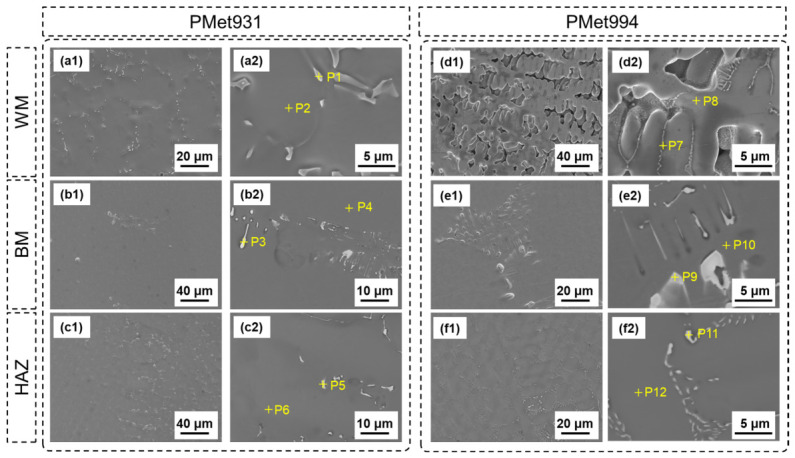
SEM microstructures of different regions in the PMet931- and PMet994-cladded specimens at different magnifications: (**a1**,**a2**) WM, (**b1**,**b2**) BM, and (**c1**,**c2**) HAZ of the PMet931-cladded specimen; (**d1**,**d2**) WM, (**e1**,**e2**) BM, and (**f1**,**f2**) HAZ of the PMet994-cladded specimen.

Figure 7 shows the SEM microstructures of different regions in the PMet931 and PMet994 cladding layers, where WM, BM, and HAZ represent the weld metal zone, base metal zone, and heat affected zone, respectively. As shown in Figure 7, both cladding layers form solidification microstructures in the weld metal zone that are distinct from the DD5 substrate, although clear differences in microstructural morphology are observed between the two systems. The weld metal zone of the PMet931 cladding layer exhibits a relatively homogeneous microstructure, with a small amount of light-colored secondary phases distributed in the matrix, locally appearing in short rod like, blocky, or network like morphologies. The base metal region retains the typical microstructural characteristics of the DD5 alloy, while a small number of precipitates and localized microstructural coarsening can be observed in the heat affected zone, indicating that the cladding thermal cycle exerts a certain influence on the near interface region of the substrate.

In contrast, the number of secondary phases in the weld metal zone of the PMet994 cladding layer increases markedly, with some regions exhibiting continuous network like or skeleton like distributions. Under higher magnification, numerous blocky, strip like, and particulate phases can be observed. This behavior is closely related to the higher W, C, and Cr contents in the PMet994 filler wire, indicating that the high concentration of carbide forming elements promotes the formation and enrichment of secondary phases in the weld metal zone. From the perspective of heat affected zone microstructures, the HAZ of the PMet931 cladded specimen shows relatively moderate microstructural changes, fewer secondary phases, and a smoother microstructural transition near the interface. In comparison, the HAZ of the PMet994 cladded specimen contains more fine precipitates and localized microstructural inhomogeneity, suggesting that PMet994 may induce stronger elemental redistribution and local microstructural evolution under the cladding thermal cycle. Owing to the higher W, C, and Cr contents in PMet994, W/Cr-rich carbides or strengthening phases are more likely to form during molten pool solidification and subsequent cooling, resulting in more pronounced secondary phase strengthening characteristics in the weld metal zone. These microstructural differences are consistent with the previously observed results that PMet994 contains a larger amount of secondary phases and exhibits higher hardness in the as-received filler wire condition.

To further analyze the elemental distribution characteristics in different regions of the cladding layers, EDS point analysis was performed on the weld metal zone, base metal zone, and heat affected zone. The results are listed in Table 3. P1–P6 correspond to different microregions in the PMet931 cladding layer, while P7–P12 correspond to different microregions in the PMet994 cladding layer. Obvious differences in elemental composition are observed between the weld metal zone and the base metal zone, indicating that elemental dilution and diffusion occurred between the cobalt-based filler wire and the DD5 substrate during cladding. The base metal characteristic regions contain relatively high contents of Ta, Ni, and Al, reflecting the compositional characteristics of the DD5 Ni based single crystal superalloy. In contrast, the contents of Co, Cr, and W increase significantly in the weld metal zone and secondary phase regions, indicating the dominant role of elements from the cobalt-based filler wires in determining the microstructure of the cladding layers.

For the PMet931 cladding layer, regions such as P1, P3, and P5 exhibit relatively high Ta contents and relatively low Ni contents, which can be mainly associated with the DD5 substrate or heat affected regions. In contrast, regions such as P2, P4, and P6 show obvious increases in Ni and Co contents, along with certain amounts of Cr, W, and Al, indicating that these regions are jointly affected by elements from both the filler wire and the substrate and exhibit clear fusion dilution characteristics. The relatively high Ni content in PMet931 helps reduce the compositional difference between the filler wire and the DD5 substrate, resulting in a comparatively smoother interfacial transition. For the PMet994 cladding layer, regions such as P7 and P8 contain high levels of Cr, W, and Co. In particular, the W content at point P7 reaches 31.95 wt%, indicating obvious W enrichment in local regions, which may correspond to W rich carbides or W rich strengthening phases. In contrast, regions such as P9 and P11 contain higher Ta and Ni contents, still showing the characteristics of the DD5 substrate or near substrate region. These results indicate that the PMet994 cladding layer exhibits a higher degree of elemental segregation and secondary phase enrichment, which is consistent with the large number of bright white secondary phases observed in SEM.

**Figure 8:**
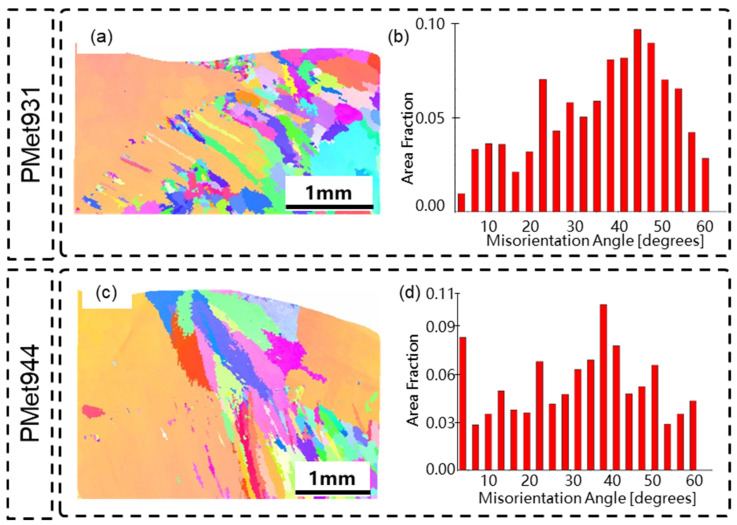
EBSD orientation maps and grain misorientation distributions of the cladding layers: (**a**,**b**) PMet931; (**c**,**d**) PMet994.

Figure 8 shows the EBSD orientation maps and grain misorientation distributions of the cross sections of the two cladding layers. Both the PMet931 and PMet994 cladding layers exhibit grain growth from the substrate toward the cladding layer, indicating that molten pool solidification during cladding is jointly influenced by the crystallographic orientation of the DD5 single crystal substrate and the direction of heat flow. The PMet931 cladding layer exhibits relatively larger grains, with some regions showing pronounced columnar grain or epitaxial growth characteristics. The grain orientations display a certain degree of continuity, suggesting a relatively good microstructural transition between the cladding layer and the DD5 substrate during solidification. Similarly, the PMet994 cladding layer also contains columnar grain regions growing along the heat flow direction. However, its grain orientation variation is more complex, and more pronounced grain refinement and orientation dispersion can be observed in local regions.

From the grain misorientation distributions, both cladding layers exhibit certain proportions of low-angle and high-angle misorientations. Here, the grain misorientation angle represents the minimum crystallographic orientation difference between adjacent grains or neighboring orientation regions, rather than an angle measured from a fixed reference direction. In this study, misorientation angles of 2–15° were defined as low-angle grain boundaries, whereas misorientation angles greater than 15° were defined as high-angle grain boundaries. These misorientation features reflect the combined effects of thermal stress, solidification shrinkage, and compositional segregation during cladding, which give rise to intragranular deformation and grain boundary misorientation differences. Compared with PMet931, the PMet994 cladding layer shows a more dispersed misorientation distribution, indicating more complex orientation variations within the microstructure. This behavior may be related to the higher W, C, and Cr contents in PMet994. On the one hand, the higher alloying element content increases the tendency for constitutional supercooling during molten pool solidification, thereby promoting competitive grain growth and local grain refinement. On the other hand, the formation of a large number of secondary phases exerts a pinning effect on grain boundary migration and grain growth, thereby altering the grain structure and orientation distribution of the cladding layer.

In summary, both PMet931 and PMet994 filler wires can form continuous cladding layers on the surface of DD5 single crystal superalloy, but their microstructural characteristics differ significantly. The PMet931 cladding layer exhibits a relatively homogeneous microstructure and a smoother interfacial transition, indicating better metallurgical compatibility. In contrast, the PMet994 cladding layer contains more secondary phases, with more pronounced local enrichment of W and Cr, showing stronger microstructural strengthening characteristics. The combined SEM, EDS, and EBSD results indicate that the higher W, C, and Cr contents in PMet994 promote the formation of W/Cr-rich secondary phases in the cladding layer and affect the solidification grain growth and orientation distribution. By contrast, the higher Ni content in PMet931 is beneficial for improving compositional compatibility and microstructural continuity with the DD5 Ni based substrate. These microstructural differences provide an important basis for explaining the subsequent differences in high-temperature tensile properties, hardness retention, and tribological behavior between the two cladding layers.

### 3.3. Mechanical Property Testing of the Cladding Layers

**Figure 9:**
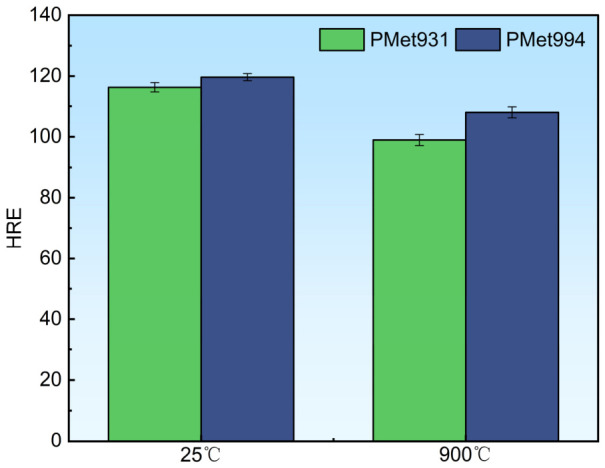
Comparison of Rockwell hardness of PMet931 and PMet994 cladding layers at room temperature and 900 °C.

To evaluate the effects of different cobalt-based filler wires on the mechanical properties of cladding layers on DD5 single crystal superalloy, room-temperature and high-temperature hardness tests were performed on the PMet931 and PMet994 cladded specimens, and tensile tests were conducted at 25, 800, 900, 1000, and 1050 °C. The results are shown in Figure 8 and Figure 9. As shown in Figure 9, both the PMet931 and PMet994 cladding layers exhibit relatively high hardness at room temperature, with values of approximately 116 HRE and 119 HRE, respectively, showing only a small difference between them. After exposure to 900 °C, the hardness of both cladding layers decreases significantly. The hardness of PMet931 decreases to approximately 99 HRE, whereas PMet994 still maintains a value of approximately 108 HRE, indicating better high-temperature hardness retention for PMet994. This behavior is mainly related to the higher W, C, and Cr contents in PMet994. As discussed in the preceding microstructural analysis, the PMet994 cladding layer contains a larger amount of W/Cr-rich secondary phases or carbide particles, which can enhance the resistance to plastic deformation through solid solution strengthening and secondary phase strengthening, while delaying microstructural softening at elevated temperatures.

**Figure 10:**
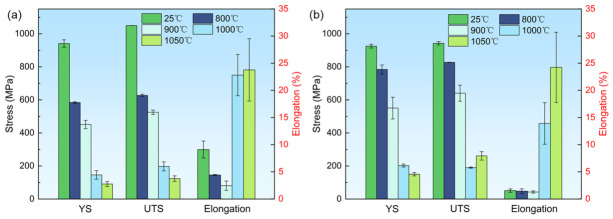
Tensile properties of DD5 cladded specimens at different temperatures: (**a**) PMet931/DD5; (**b**) PMet994/DD5.

As shown in Figure 10, with increasing test temperature, the ultimate tensile strength and yield strength of both cladded specimens decrease overall, while the elongation first decreases and then increases. At room temperature, the ultimate tensile strength, yield strength, and elongation of PMet931 are approximately 1019 and 941 MPa, and 7.9%, respectively, demonstrating a good balance between strength and ductility. In contrast, the ultimate tensile strength and yield strength of PMet994 are approximately 942 MPa and 925 MPa, respectively, while its elongation is only about 1.5%. These results indicate that PMet931 exhibits better ductility and overall mechanical performance at room temperature, which may be related to its higher Ni content that improves the compositional compatibility and interfacial metallurgical compatibility with the DD5 Ni based substrate. When the temperature increases to 800 °C and above, the high-temperature strength advantage of PMet994 gradually becomes apparent. At 800 °C, the ultimate tensile strength and yield strength of PMet994 are approximately 827 MPa and 784 MPa, respectively, significantly higher than the corresponding values of 658 MPa and 617 MPa for PMet931. At 900 °C, the ultimate tensile strength and yield strength of PMet994 are approximately 583 MPa and 496 MPa, respectively, remaining higher than those of PMet931, which are approximately 525 MPa and 451 MPa. With further temperature increase to 1000 °C and 1050 °C, the strengths of both specimens continue to decrease; however, PMet994 still maintains a higher strength level. At 1050 °C, the ultimate tensile strength and yield strength of PMet994 are approximately 190 MPa and 150 MPa, respectively, whereas the corresponding values for PMet931 are approximately 124 MPa and 91 MPa. These results demonstrate that the PMet994 cladding layer possesses stronger high-temperature load bearing capability and greater resistance to softening.

In terms of elongation, both cladded specimens exhibit relatively low values in the temperature range of 800–900 °C, with PMet994 showing overall lower elongation than PMet931, indicating that its higher hardness and strength are accompanied by a certain loss of ductility. This behavior may be related to the larger amount of secondary phases and more pronounced local elemental segregation in the PMet994 cladding layer. Hard phases and compositionally inhomogeneous regions are prone to generating stress concentrations during tensile deformation. As the temperature increases above 1000 °C, the elongation of both specimens increases significantly. At 1050 °C, the elongation values of PMet931 and PMet994 reach approximately 23.7% and 24.2%, respectively, indicating enhanced plastic deformation capability at elevated temperatures and improved deformation compatibility between the interface and the cladding layer.

Overall, the PMet931 cladded specimen exhibits a better balance between strength and ductility at room temperature, whereas the PMet994 cladded specimen shows superior strength and hardness retention over the temperature range of 800–1050 °C. The differences in performance mainly originate from the differences in filler wire composition and strengthening mechanisms. The higher Ni content in PMet931 is beneficial for improving its metallurgical compatibility with the DD5 substrate, while the higher W, C, and Cr contents in PMet994 promote the formation of W/Cr-rich secondary phases, thereby enhancing solid solution strengthening and secondary phase strengthening effects, which ultimately improve the high-temperature strength and resistance to softening of the cladding layer.

### 3.4. High-Temperature Tribological Properties of the Cladding Layers

**Figure 11:**
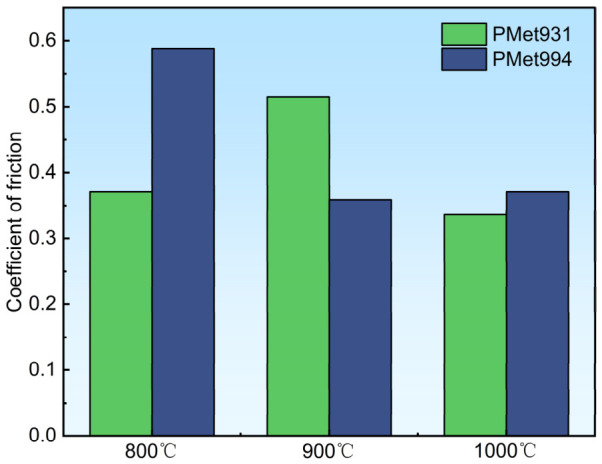
Coefficients of friction of the cladding layers at different temperatures.

**Figure 12:**
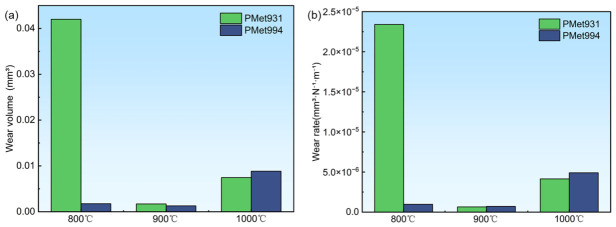
Wear volume and wear rate of the cladding layers at different temperatures: (**a**) wear volume; (**b**) wear rate.

To evaluate the wear resistance of the two cobalt-based cladding layers under high-temperature conditions, high-temperature tribological tests were conducted on the PMet931 and PMet994 cladded specimens at 800 °C, 900 °C, and 1000 °C. The results are shown in Figure 11 and Figure 12. As shown in Figure 11, the coefficients of friction of the two cladding layers exhibit different temperature-dependent trends. At 800 °C, the COF of PMet994 is approximately 0.588, which is higher than that of PMet931 of approximately 0.371, indicating greater interfacial frictional resistance between the PMet994 cladding layer and the silicon nitride counterface under this condition. When the temperature increases to 900 °C, the COF of PMet931 increases to 0.515, whereas that of PMet994 decreases to 0.359, showing opposite variation trends for the two materials. At 1000 °C, the coefficients of friction of the two cladding layers become comparable, with values of approximately 0.336 and 0.371 for PMet931 and PMet994, respectively. These results indicate that, during high-temperature sliding, the COF is governed not only by hardness but also by the formation and stability of the surface oxide films, the debris compaction behavior, and the interfacial contact state.

Figure 12 shows the wear volumes and wear rates of the two cladding layers at different temperatures. At 800 °C, the wear volume and wear rate of PMet931 are 4.20 × 10^−2^ mm^3^ and 2.34 × 10^−5^ mm^3^·N^−1^·m^−1^, respectively, whereas those of PMet994 are only 1.76 × 10^−3^ mm^3^ and 9.76 × 10^−7^ mm^3^·N^−1^·m^−1^, respectively. This indicates that PMet994 exhibits a much lower material removal rate and superior wear resistance at 800 °C, although its COF is higher than that of PMet931.

The apparently opposite trends in COF and wear rate at 800 °C can be explained by the different physical meanings of these two parameters. The COF mainly reflects the interfacial shear resistance and contact state during sliding, whereas the wear rate is more directly related to the actual material removal from the worn surface. For the PMet994 cladding layer, the higher W, C, and Cr contents promote the formation of W/Cr-rich secondary phases and improve the hardness and resistance to softening. These hard secondary phases can effectively resist ploughing, cutting, and plastic deformation during sliding, thereby reducing the wear volume and wear rate. However, the presence of more hard secondary phases and local microstructural inhomogeneity may increase the mechanical interlocking and interfacial shear resistance with the silicon nitride counterface. In addition, the oxide film formed at 800 °C may not be sufficiently continuous or lubricative. As a result, PMet994 shows a higher COF but a lower wear rate than PMet931 at 800 °C.

When the temperature increases to 900 °C, the wear loss of both cladding layers decreases significantly. The wear rates of PMet931 and PMet994 are 6.52 × 10^−7^ and 7.15 × 10^−7^ mm^3^·N^−1^·m^−1^, respectively, showing only a small difference. These results suggest that a relatively continuous and dense oxide film may form on the friction surface at 900 °C, providing a protective effect and reducing the material removal. At 1000 °C, the wear loss of both cladding layers increases again. The wear rates of PMet931 and PMet994 are 4.14 × 10^−6^ and 4.91 × 10^−6^ mm^3^·N^−1^·m^−1^, respectively, indicating that the protective effect of the oxide film decreases at excessively high temperatures. Consequently, surface softening, oxide film instability, and intensified oxidative wear lead to increased material removal.

Overall, the PMet994 cladding layer exhibits significantly lower wear volume and wear rate at 800 °C, indicating that secondary phase strengthening plays an important role in improving wear resistance at medium-to-high temperatures. However, in the temperature range of 900–1000 °C, the difference in wear rate between the two cladding layers decreases, and PMet994 even shows a slightly higher wear rate than PMet931. This indicates that high-temperature wear behavior is not governed solely by hardness. For cobalt-based cladding layers on the DD5 surface, high-temperature wear resistance is jointly controlled by compositional strengthening, secondary phase distribution, oxide film stability, surface softening, and damage mechanisms at the frictional interface. Therefore, PMet994 shows a pronounced wear resistance advantage at 800 °C due to its higher hardness and stronger secondary phase strengthening, whereas PMet931 exhibits relatively stable wear behavior at higher temperatures due to its smoother microstructural transition and better interfacial compatibility.

### 3.5. Composition, Microstructure and Property Relationships

Based on the above results, PMet931 and PMet994 cobalt-based filler wires exhibit distinctly different microstructural characteristics and high-temperature properties after cladding on the surface of DD5 single crystal superalloy. The fundamental reason for these differences lies in their different alloy design strategies. PMet931 contains a higher Ni content, whereas PMet994 contains significantly higher levels of W, C, and Cr. Ni exhibits good compositional compatibility with the DD5 Ni based single crystal superalloy substrate, which is beneficial for reducing the degree of abrupt compositional change between the filler wire and the substrate, thereby promoting a relatively smooth microstructural transition between the cladding layer and the substrate. Consequently, the PMet931 cladded specimen exhibits higher ultimate tensile strength and better ductility at room temperature, indicating superior interfacial compatibility and a better overall balance between strength and ductility.

In contrast, the higher W, C, and Cr contents in PMet994 significantly enhance the strengthening effect of the cladding layer. On the one hand, W improves the high-temperature strength and resistance to softening of the Co-based matrix through solid solution strengthening. On the other hand, C readily combines with elements such as W and Cr to form W/Cr-rich carbides or secondary phase particles, which contribute to dispersion strengthening and grain boundary pinning within the cladding layer. The SEM and EDS results indicate that the PMet994 cladding layer contains a larger amount of secondary phases, with more pronounced local enrichment of W and Cr. The EBSD results further show more complex grain orientation variations and more prominent microstructural strengthening characteristics. Consequently, PMet994 maintains higher hardness at 900 °C and exhibits higher ultimate tensile strength and yield strength over the temperature range of 800–1050 °C.

However, an increased amount of strengthening phases does not necessarily lead to simultaneous improvement in ductility and wear stability over the entire temperature range. Although the larger number of hard secondary phases and local elemental segregation in the PMet994 cladding layer enhance hardness and high-temperature strength, they also tend to generate stress concentrations during tensile deformation, resulting in lower elongation than PMet931 at room temperature and in the temperature range of 800–900 °C. The tribological results likewise demonstrate that the wear rate of PMet994 at 800 °C is significantly lower than that of PMet931, reflecting the positive role of hard phase strengthening in improving resistance to ploughing and abrasive wear. However, at 900 °C and 1000 °C, the difference in wear rate between the two cladding layers decreases markedly, indicating that high-temperature wear behavior is governed not only by hardness but also by the combined effects of oxide film formation, debris compaction, surface softening, and interfacial stability.

Therefore, the effects of the two filler wires on the properties of DD5 cladding layers can be summarized as two distinct strengthening pathways. PMet931 primarily relies on its higher Ni content to improve metallurgical compatibility with the DD5 substrate, enabling the cladding layer to achieve better room-temperature strength ductility balance and microstructural compatibility. In contrast, PMet994 relies on its high W, high C, and relatively high Cr contents to produce stronger solid solution strengthening and secondary phase strengthening, thereby providing the cladding layer with higher hardness, superior high-temperature strength, and better wear resistance at 800 °C. If service conditions place greater emphasis on ductility matching and interfacial reliability at room temperature or medium to low temperatures, PMet931 is more advantageous. In contrast, if the service environment is dominated by high-temperature load bearing, resistance to softening, and high-temperature wear resistance, PMet994 exhibits greater application potential.

In summary, the composition of cobalt-based filler wires influences the hardness, high-temperature tensile properties, and tribological behavior of cladding layers on DD5 single crystal superalloy by regulating elemental diffusion, secondary phase precipitation, grain structure, and interfacial microstructural characteristics within the cladding layer. Among the alloying elements, Ni content mainly affects the compositional compatibility and ductility matching between the filler wire and the Ni based substrate, whereas W, C, and Cr contents primarily determine the degree of secondary phase strengthening and the retention of high-temperature properties. These results indicate that, for cladding on the surface of DD5 single crystal superalloy, cobalt-based filler wire compositions should be selected according to the actual service temperature and performance requirements in order to achieve a balanced combination of strength, ductility, and wear resistance in the cladding layer.

## 4. Conclusions

This study addresses the selection of suitable cobalt-based filler wires for cladding on DD5 single crystal superalloy, where interfacial compatibility, high-temperature strength, and wear resistance must be balanced. By comparing PMet931 and PMet994 cladding layers, the effects of filler wire composition on microstructure, hardness, high-temperature tensile properties, and tribological behavior were clarified. The results provide guidance for filler wire selection and performance optimization of cladding layers on Ni-based single crystal superalloy components. The main conclusions are as follows:

(1) The alloy design strategies of PMet931 and PMet994 differ significantly. PMet931 contains higher Ni, whereas PMet994 contains higher W, C, and Cr. The initial hardness of PMet994 is approximately 24% higher than that of PMet931, indicating stronger solid solution strengthening and secondary phase strengthening.

(2) Both filler wires form continuous cladding layers on the DD5 substrate. PMet931 produces a relatively homogeneous microstructure and smoother interfacial transition, whereas PMet994 promotes the formation of more W/Cr-rich secondary phases, local elemental enrichment, and grain refinement.

(3) PMet931 exhibits better room-temperature strength ductility balance, which is related to improved interfacial compatibility induced by its higher Ni content. In contrast, PMet994 shows higher strength and better hardness retention at 800–1050 °C, with the ultimate tensile strength at 1050 °C being approximately 53% higher than that of PMet931.

(4) PMet994 exhibits a significantly lower wear rate than PMet931 at 800 °C, mainly due to its higher hardness and W/Cr-rich secondary phase strengthening. However, at 900–1000 °C, the wear rate difference decreases, indicating that the high-temperature wear behavior is jointly controlled by hardness, oxide film stability, and frictional interface damage.

## Figures and Tables

**Table 1:** Chemical compositions of the DD5 substrate and PMet931 and PMet994 cobalt-based filler wires (wt%).

Element	Cr	W	Ni	C	Mn	Si	Fe	Mo	Al	Ta	Hf	Re	Co
PMet931	24.50	7.37	10.61	0.46	0.66	0.64	1.52	/	/	/	/	/	Bal.
PMet994	27.82	18.8	5.40	0.95	0.24	0.64	2.68	/	/	/	/	/	Bal.
DD5	7.02	5.08	Bal.	0.05	/	/	/	1.53	6.21	6.58	0.15	2.67	7.55

**Table 2:** EDS point analysis results of different regions in Figure 4 (wt%).

Test Points	Chemical Compositions (wt%)						
	Si	W	Cr	Fe	Co	Ni	V
P1	0.27	3.23	76.06	0.44	17.96	2.03	-
P2	0.65	24.11	1.8	56.35	10.56	6.53	-
P3	0.87	24.77	2.03	55.68	10.97	5.68	-
P4	4.2	65.07	1.71	20.44	3.49	5.09	-
P5	1.05	11.38	25.75	3.35	50.72	6.34	1.41
P6	1.29	11.73	34.41	3.13	43.11	4.98	1.35
P7	0.87	16.27	30.21	2.82	44	4.61	1.22

**Table 3:** EDS point analysis results for different regions in Figure 7 (wt%).

Test Points	Chemical Compositions (wt%)								
	Al	Si	Mo	Cr	Fe	Co	Ni	Ta	W
P1	2.63	0	2.83	4.09	0.86	4.16	5.18	70.05	10.20
P2	5.75	0	1.65	10.88	0.76	25.31	42.01	4.14	9.51
P3	3.25	0	4.06	1.25	0.38	1.58	7.72	74.82	6.93
P4	9.52	0	1.86	5.25	0	6.60	62.92	6.26	7.60
P5	0.89	0	1.11	3.19	0.09	3.23	7.33	75.00	9.16
P6	9.44	0	1.67	5.38	0	8.43	61.88	4.13	9.08
P7	2.48	0	3.88	29.67	0.70	12.88	13.50	4.94	31.95
P8	6.31	0	1.52	12.25	1.15	23.72	40.23	2.60	12.23
P9	3.03	0	3.58	1.14	0.65	1.55	5.62	76.61	7.72
P10	9.71	0	2.37	5.35	0.04	6.58	61.17	6.83	7.94
P11	3.14	0	3.99	2.35	0	2.40	9.21	69.20	9.71
P12	9.01	0	2.07	8.31	0.43	12.37	53.02	5.14	9.64

## Data Availability

The original contributions presented in this study are included in the article. Further inquiries can be directed to the corresponding authors.
